# A large population-based investigation into the genetics of susceptibility to gastrointestinal infections and the link between gastrointestinal infections and mental illness

**DOI:** 10.1007/s00439-020-02140-8

**Published:** 2020-03-09

**Authors:** Ron Nudel, Vivek Appadurai, Andrew J. Schork, Alfonso Buil, Jonas Bybjerg-Grauholm, Anders D. Børglum, Mark J. Daly, Ole Mors, David M. Hougaard, Preben Bo Mortensen, Thomas Werge, Merete Nordentoft, Wesley K. Thompson, Michael E. Benros

**Affiliations:** 1 Institute of Biological Psychiatry, Mental Health Centre Sct. Hans, Mental Health Services Copenhagen, Roskilde, Denmark; 2 iPSYCH, The Lundbeck Foundation Initiative for Integrative Psychiatric Research, Copenhagen, Denmark; 3 Department for Congenital Disorders, Center for Neonatal Screening, Statens Serum Institute, Copenhagen, Denmark; 4 Department of Biomedicine, Aarhus University and Centre for Integrative Sequencing, iSEQ, Aarhus, Denmark; 5 Aarhus Genome Center, Aarhus, Denmark; 6 Stanley Center for Psychiatric Research, Broad Institute of Harvard and MIT, Cambridge, MA USA; 7 Psychosis Research Unit, Aarhus University Hospital, Risskov, Denmark; 8 National Center for Register-Based Research, Aarhus University, Aarhus, Denmark; 9 Department of Clinical Medicine, Faculty of Health and Medical Sciences, University of Copenhagen, Copenhagen, Denmark; 10 Mental Health Centre Copenhagen, Copenhagen University Hospital, Kildegaardsvej 28, Entrance 15, 4th floor, 2900 Hellerup, Denmark; 11 Department of Family Medicine and Public Health, Division of Biostatistics, University of California, San Diego, CA USA

## Abstract

Gastrointestinal infections can be life threatening, but not much is known about the host’s genetic contribution to susceptibility to gastrointestinal infections or the latter’s association with psychiatric disorders. We utilized iPSYCH, a genotyped population-based sample of individuals born between 1981 and 2005 comprising 65,534 unrelated Danish individuals (45,889 diagnosed with mental disorders and 19,645 controls from a random population sample) in which all individuals were linked utilizing nationwide population-based registers to estimate the genetic contribution to susceptibility to gastrointestinal infections, identify genetic variants associated with gastrointestinal infections, and examine the link between gastrointestinal infections and psychiatric and neurodevelopmental disorders. The SNP heritability of susceptibility to gastrointestinal infections ranged from 3.7% to 6.4% on the liability scale. Significant correlations were found between gastrointestinal infections and the combined group of mental disorders (OR = 2.09; 95% CI: 1.82–2.4, *P* = 1.87 × 10^–25^). Correlations with autism spectrum disorder, attention deficit hyperactivity disorder, and depression were also significant. We identified a genome-wide significant locus associated with susceptibility to gastrointestinal infections (OR = 1.13; 95% CI: 1.08–1.18, *P* = 2.9 × 10^–8^), where the top SNP was an eQTL for the *ABO* gene. The risk allele was associated with reduced *ABO* expression, providing, for the first time, genetic evidence to support previous studies linking the O blood group to gastrointestinal infections. This study also highlights the importance of integrative work in genetics, psychiatry, infection, and epidemiology on the road to translational medicine.

**Electronic supplementary material:**

The online version of this article (10.1007/s00439-020-02140-8) contains supplementary material, which is available to authorized users.

## Introduction

Gastrointestinal infections are a leading cause of hospitalizations and can be fatal in children under the age of five, even in developed countries (Fischer et al. 2007). Gastrointestinal infections spread not only from person to person, but also through contact with a contaminated environment (Musher and Musher 2004). Both the genetics of the host and the host’s environment are believed to influence susceptibility to acquiring an infection (Chapman and Hill 2012; Patarcic et al. 2015). However, not much is known about the genetic architecture of susceptibility to gastrointestinal infections or to what degree they may be associated with mental disorders, which could be of interest, as the former have been linked to alterations in central nervous system function and behavior (Cryan and Dinan 2012).

With regards to this latter point, gastrointestinal infections can also influence the composition of the gut microbiome, and an increasing number of studies point to a link between the gut microbiome and the brain, with which it “communicates” via the vagus nerve, immune mediators or metabolites produced by bacteria (Sherwin et al. 2016). Furthermore, the gut microbiome and the gastrointestinal tract have been implicated in several psychiatric and neurodevelopmental disorders: it has been shown that patients with schizophrenia may exhibit gastrointestinal dysfunction and have structural damage to the gastrointestinal tract resulting from pathogens (Nemani et al. 2015). Moreover, there have been a multitude of reports indicating comorbidity between schizophrenia and gastrointestinal inflammation (Severance et al. 2015). A link between the gut microbiome and depression has also been reported (Valles-Colomer et al. 2019). In the case of autism spectrum disorder (ASD), comorbidities between ASD and gastrointestinal disorders are commonly reported, with the degree of comorbidity often correlating with the severity of the autistic behavior (Coury et al. 2012; Hsiao 2014). The gut microbiome, specifically, has also been an object of study in the context of ASD, with the levels of certain bacteria being significantly higher or lower in autistic children compared to controls (Adams et al. 2011; Parracho et al. 2005). Similar results were reported for attention deficit hyperactivity disorder (ADHD) as well (Aarts et al. 2017). Interestingly, one study reported a genetic risk factor specific to a comorbid diagnosis of ASD and gastrointestinal dysfunction (Campbell et al. 2009). A gastrointestinal infection caused by a specific pathogen, *H. pylori,* has also been shown to be associated with changes in cognitive function and behavior (Budzynski and Klopocka 2014).

Given such compelling evidence for the connection between the gastrointestinal tract and psychiatric and neurodevelopmental disorders, which are known to be quite heritable (Schork et al. 2019b), and given the importance of understanding the genetic basis of gastrointestinal infections to elucidate the disease mechanism on the path to better diagnosis and treatment, we sought to investigate gastrointestinal infections from both angles outlined above: the genetic architecture of susceptibility to gastrointestinal infections, and their links with psychiatric and neurodevelopmental disorders. To achieve this, we used a population-based Danish cohort of 65,534 individuals from the Integrative Psychiatric Research (iPSYCH) initiative (Pedersen et al. 2017), chosen either for having at least one of the following psychiatric or neurodevelopmental disorders: ASD, ADHD, schizophrenia, bipolar disorder, depression and anorexia, or as part of a random population sample representative of the Danish population and not selected for mental disorders or infections. All individuals’ records were then assessed for infections requiring hospitalization. We have recently reported a strong association between a general infection phenotype and psychiatric disorders in the iPSYCH cohort (Nudel et al. 2019), and in this study, we extend our investigation to gastrointestinal infections in particular. Our analyses here include a genetic assessment of gastrointestinal infection in the form of SNP heritability and a genome-wide association study, as well as comorbidity analyses to find potential links between gastrointestinal infection and the six main iPSYCH disorders.

## Materials and methods

### Data sources

Data were obtained by linking Danish population-based registers using the unique personal identification number, which is assigned to all live-born children and new residents in Denmark since 1968 and used across all registration systems (Pedersen 2011). The Danish Civil Registration System contains records of births, deaths, immigration, emigration, and links to family members. The Danish Neonatal Screening Biobank stores dried blood spots taken at birth from nearly all infants born in Denmark after 1981 (Norgaard-Pedersen and Hougaard 2007; Pedersen 2011). Information about infections was obtained from the Danish National Hospital Registry, which, since 1977, has contained records of all inpatients treated at Danish non-psychiatric hospitals, and, from 1995 and onwards, has contained information regarding outpatient and emergency room contacts (Andersen et al. 1999). The Psychiatric Central Research Registry covers all psychiatric inpatient facilities since 1969 as well as outpatient contacts since 1995 (Mors et al. 2011). For both registries, diagnostic information was based on the 8th Revision of the International Classification of Diseases (ICD-8) (World Health Organization 1971) from 1977 to 1993, and on the 10th Revision (ICD-10) from 1994 (World Health Organization 1994).

### Assessment of psychiatric disorders

All singletons born in Denmark between May 1, 1981 and December 31, 2005, who were residents in Denmark on their first birthday and who have a known mother (*n* = 1,472,762) were considered. From this group, 86,189 individuals were included in the original study sample (Pedersen et al. 2017). Before quality control (QC), our sample included 78,050 successfully genotyped individuals. Following QC, 65,534 individuals remained: 19,645 individuals with no hospital contacts for psychiatric diagnoses (ICD-10: F00–F99), and 45,889 individuals mostly with one or more of the following mental disorders: ASD (F84.0–1, F84.5, F84.8–9, *N* = 12,331), ADHD (F90.0, *N* = 14,397), schizophrenia (F20, *N* = 2401), bipolar disorder (F30–F31, *N* = 1391), anorexia nervosa (F50.0, *N* = 2551), and single and recurrent depressive disorder (F32–F33, *N* = 18,511). These diagnoses are based on data from the Danish Psychiatric Central Research Register only. A minority of the individuals with a psychiatric diagnosis (*N* = 1993), most of whom were originally included as part of the random population sample, had other ICD-10 chapter V diagnoses. Individual-based data were available until an individual’s emigration, death, or until 31 December 2012.

### Assessment of infections

All infection cases requiring hospital contact were identified in the Danish National Hospital Registry. All hospital contacts for infections were included with ICD-8 and ICD-10 codes listed in Supplementary Table S1, as used in previous studies (Benros et al. 2013, 2015, 2016; Nudel et al. 2019), and each person may have had a history of more than one infection. We omitted all diagnoses with the modification code “suspected” or “not found”. In the current study, there were 7197 individuals with at least one gastrointestinal infection, and 37,062 individuals without any of the infection categories identified in the iPSYCH sample (bacterial, viral, CNS, gastrointestinal, genital, hepatitis, otitis, pregnancy related (infection in the mother while pregnant with the child who is in iPSYCH), respiratory, sepsis, skin infection, urological, or other).

### Defining cases and controls

Cases were defined as having the relevant ICD code (for psychiatric diagnoses) or one of several codes in the gastrointestinal infections category (for gastrointestinal infections) as per the above criteria. There is very high comorbidity among the psychiatric cases in the iPSYCH cohort (Schork et al. 2019b), and, for gastrointestinal infections, around 93% of the people who had gastrointestinal infections were also diagnosed with at least one other infection category from the previous section. It has been shown that individuals with one psychiatric diagnosis are more prone to getting another one, and this higher “comorbidity risk” can persist over time, as reported for a much larger Danish dataset (Plana-Ripoll et al. 2019). We, therefore, adopted two approaches: in one approach, controls cannot have any psychiatric diagnosis or any infection category for their respective phenotype (super controls); for example, in the genome-wide association study (GWAS) for gastrointestinal infections, we excluded individuals who had other infection categories from being controls. This could result in population-biased effect size estimates, but it also provides more power and does not inflate type I error. The second approach used as “normal controls” individuals who did not have the diagnosis in question, irrespective of whether they had other psychiatric diagnoses or infection categories, where appropriate. As the comorbidity and (especially) the heritability analyses could be more affected by extreme sampling (Schork et al. 2019a; Yap et al. 2018), we repeat those analyses using normal controls as well. Table 1 specifies which subsets were used in which analyses.

**Table 1:** A summary of which of the various subsets were used across all analyses

Analysis	Entire sample with super controls and a covariate for psychiatric diagnosis	Entire sample with normal controls and a covariate for psychiatric diagnosis	Random population sample with super controls	Random population sample with normal controls
Comorbidity analysis			×	×
Heritability estimation	×	×	×	×
GWAS	×			

Some analyses were performed several times, with a different subset each time

### Genetic markers, quality control for markers and samples, and imputation

Samples were genotyped on the Illumina Psych chip. Before quality control (QC), there were 78,050 samples genotyped in 23 waves from the original sample. A full description of the procedure of the sample and SNP QC is provided elsewhere (Schork et al. 2019b). Briefly, a principal component analysis (PCA) was performed using the iPSYCH sample with 1000 Genomes Project samples as a reference panel to compute the initial principal component space. Individuals whose parents and grandparents were born in Denmark were used as a reference in removing individuals who were a certain distance from the multivariate mean of the joint distribution of the first 10 PCs. This was then repeated using only the iPSYCH sample to identify subtler within-population differences. Samples were also removed based on genotype missingness, abnormal heterozygosity or ambiguous sex, based on genetic markers. Samples that were identified as duplicates were also removed. Lastly, samples that were found to be related to other samples (first and second degree) were removed, whereby the cases and then samples with a higher genotype call rate were prioritized. Following this, a new PCA was performed to obtain principal components for downstream analyses. For the imputation, only autosomal SNPs were used, and SNPs were removed based on low minor allele frequency, Hardy–Weinberg equilibrium *P* value, having more than two alleles, or being indels. Genotypes were phased with SHAPEIT3 (O'Connell et al. 2016) and imputed with IMPUTE2 (Howie et al. 2009). Imputed markers were removed if they had an INFO score below 0.2, a minor allele frequency (MAF) below 0.001, best-guess genotypes missing in > 10% of subjects, Hardy–Weinberg equilibrium (HWE) *P* < 1 × 10^–6^ (in controls) or a highest probability for a genotype of less than 0.9. Markers were also removed if they were significantly associated with genotyping wave. Two marker datasets were used: in the GWAS, all post-QC dosage data were used. This dataset included 11,600,722 markers. For the heritability and PRS analyses, which are based on an aggregation of SNPs, the above dataset was filtered, resulting in a dataset of high-confidence imputations (best-guess genotypes), with markers having an INFO score of at least 0.8 and MAF of at least 0.01. This dataset included 7,071,055 markers. Positions throughout this paper are in hg19.

### Statistical analysis

#### Comorbidity analysis

A logistic regression analysis was performed with the glm function in R (R Core Team 2014) v3.3.1 to examine the association between having ASD, ADHD, schizophrenia, bipolar disorder, depression, anorexia or phenotype comprising any psychiatric diagnosis (F00–F99) as the outcome and having gastrointestinal infections as the independent variable. These analyses included covariates for age, age squared and sex, and used only QC-passing individuals from the random population sample. Two sets of analyses were performed: one with super controls and one with normal controls. The number of individuals with both a gastrointestinal infection diagnosis and a psychiatric diagnosis in the random population sample was not large (as reflected in the wide confidence intervals), but this allowed us to avoid potential biases resulting from the selection of psychiatric cases for the iPSYCH cohort.

#### SNP heritability

GCTA (Yang et al. 2011) v1.91.1 beta (or v1.91.4 beta in later analyses) was used to compute the SNP heritability for susceptibility to gastrointestinal infections. Genetic relationship matrices were calculated for each autosomal chromosome separately with --make-grm and merged with --mgrm. Four GREML analyses were performed: with super controls or normal controls, and in the entire sample (with a covariate for having any psychiatric diagnosis) or only with QC-passing individuals from the random population sample. The latter analyses had reduced sample size and power and were performed to check for any bias given the enrichment of infections in psychiatric cases, who comprised the majority of the iPSYCH sample. All analyses were performed with covariates of age, age squared, sex, and the first ten PCs.

#### Genome-wide association study of susceptibility to gastrointestinal infection

PLINK (Purcell et al. 2007) v1.90b3.34 was used for performing a logistic regression with covariates for age, age squared, sex, the first ten PCs, and a covariate for having a psychiatric diagnosis (ICD-10 codes F00–F99). This was done to account for the observed correlation between the infection phenotype and the psychiatric phenotype, as shown in the comorbidity analyses. The GWAS used super controls, but, to make sure the associated locus was not associated with susceptibility to general infection, the effect sizes of the top SNP from this GWAS and a GWAS which used any infection category as its outcome were compared using a *Z* test (Gelman and Stern 2006). Since the two subsets had overlapping controls, we bootstrapped the difference between the two estimates using the R package boot (Canty and Ripley 2017; Davison and Hinkley 1997) (with *R* = 10,000) and replaced the denominator of the *Z* score with the standard deviation of the distribution of estimate differences.

## Results

In the sample of 65,534 Danish unrelated individuals born after 1981, a total of 28,472 individuals had infections requiring hospitalization during the study period from birth to end of follow-up, of whom a total of 7197 individuals had gastrointestinal infections. Among the 45,889 individuals with a psychiatric diagnosis, a total of 21,728 individuals had hospitalizations for infections, of whom 5640 were diagnosed with gastrointestinal infections. Among the 19,645 individuals with no psychiatric diagnosis, a total of 6744 individuals had hospitalizations for infections, among them 1557 had gastrointestinal infections. Among the 21,706 individuals in the post-QC random population sample, 1851 had a diagnosis of gastrointestinal infection, 2175 had a psychiatric diagnosis, and 303 had both.

### Comorbidity analysis

**Fig. 1:**
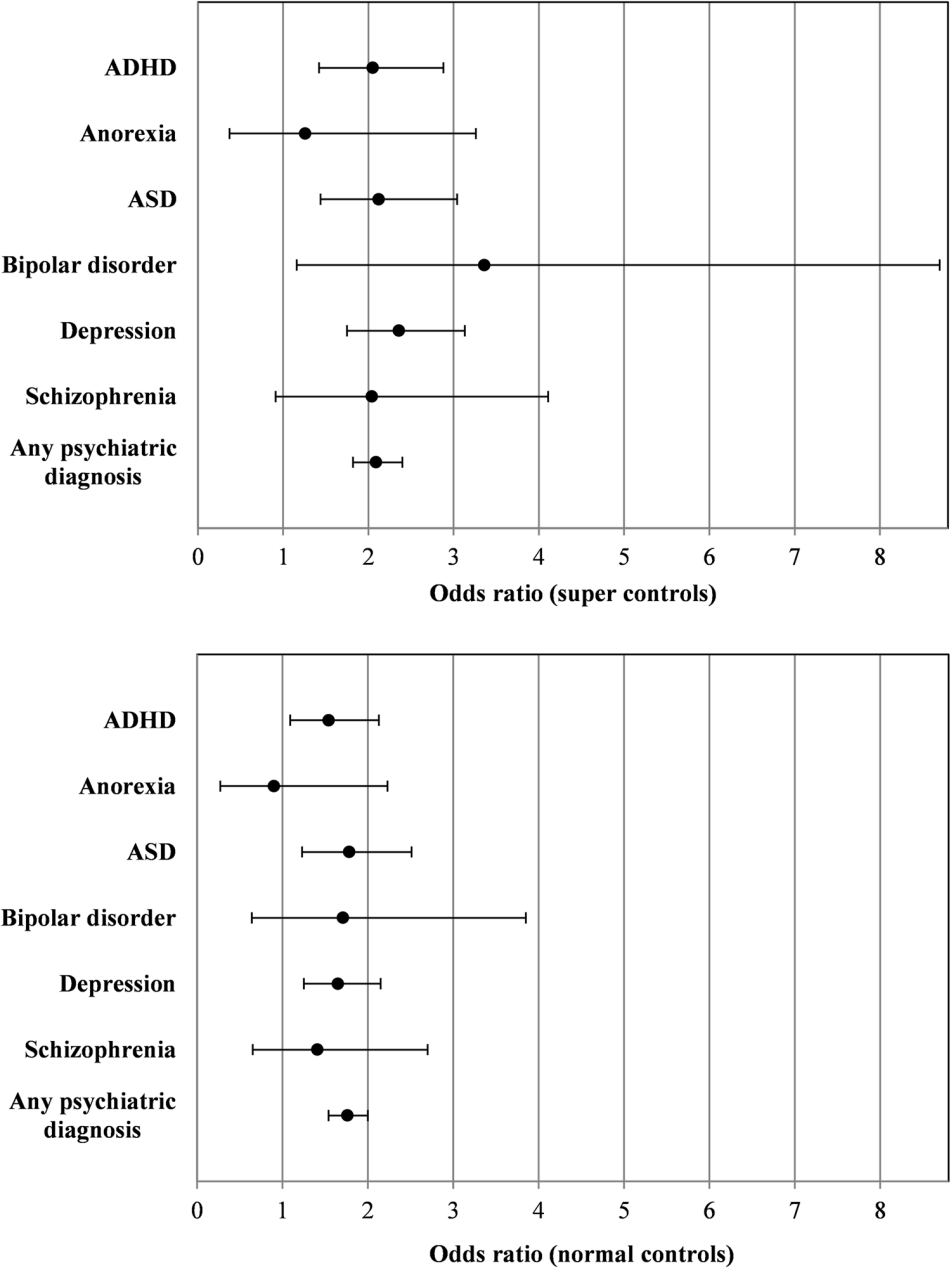
Comorbidity analyses employing logistic regression of the psychiatric diagnosis on the diagnosis of gastrointestinal infection

In the regression analyses, when using super controls, all odds ratios (ORs) for the regressions of the psychiatric diagnoses on gastrointestinal infection status were significantly larger than unity, except for the ORs for anorexia and schizophrenia. The association between gastrointestinal infections and any psychiatric disorder (ICD-10 codes F00–F99) was highly significant [OR = 2.09; 95% confidence interval (CI): 1.82–2.4, *P* = 1.87 × 10^–25^]. In this model, the age covariates were also significantly associated with the psychiatric outcome; however, both the model which used super controls and the one which used normal controls improved by the addition of the gastrointestinal infection diagnosis as a variable, as indicated by a likelihood ratio test for both approaches using nested models (maximum *P* = 2.546 × 10^–15^). The individual associations with ASD, ADHD, and depression were all significant after Bonferroni correction (*P* = 7.28 × 10^–5^, 6.20 × 10^–5^, 6.60 × 10^–9^, respectively). When using normal controls, the association with any psychiatric disorder remains significant (OR = 1.76; 95% CI: 1.54–2, *P* = 1.07 × 10^–16^), but otherwise only the associations with ASD (*P* = 0.0015) and depression (*P* = 3.05 × 10^–4^) remain significant after Bonferroni correction (14 tests in total with both approaches). Figure 1 presents ORs and 95% CIs for all analyses. It should be mentioned that individuals diagnosed with mental illness could potentially be more likely to receive a diagnosis of gastrointestinal infection than individuals without mental illness due to the fact that the former are already in hospital contact. While this is hypothetically possible and could drive the association between psychiatric diagnoses and infection diagnoses, we observe that the majority of individuals with a comorbid diagnosis in our sample received the infection diagnosis first: we defined a variable which was the age at the gastrointestinal infection diagnosis minus the age at the first psychiatric diagnosis (in years) for individuals with both types of diagnosis. In the random population sample, in which the above regressions were performed, the distribution of this variable had a mean of − 7.74, a median of − 7.23, and a standard deviation of 8.24. This implies that, on average, individuals received the psychiatric diagnosis almost 8 years after the infection diagnosis, and that more than 50% of the individuals in question received the infection diagnosis first. Similar values were observed even when taking into account all comorbid individuals, not only from the random population sample.

### Heritability estimation

In the entire sample, when using super controls, the SNP heritability on the observed scale was a moderate but statistically significant 3.5% (SE = 0.007, *P* = 1.09 × 10^–7^). To transform the observed heritability to the liability scale, one needs to know the lifetime prevalence of the disease *k* and the proportion of cases in the sample used to estimate the SNP heritability (0.163 in this case) (Lee et al. 2011). The prevalence of gastrointestinal infections in the random population sample was 8.5%, which means that the minimum *k* value is 8.5%. This translates to a heritability of 6.4% on the liability scale. When using normal controls, the SNP heritability on the observed scale was lower at 1.6% (SE = 0.005, *P* = 0.000247). With a *k* of 8.5% and a proportion of cases of 0.11, this translates to a heritability of 4.1% on the liability scale. These analyses were repeated using only individuals from the random population sample. While these analyses had a much reduced sample size, they provide population-unbiased heritability estimates, unaffected by enrichment of infection cases among psychiatric the cases in the cohort. Although the estimates from these analyses were not significant, they were close to the one reported above: with super controls, the heritability on the observed scale was 1.5% (SE = 0.018, *P* = 0.199), translating to 3.7% on the liability scale (the proportion of cases was 0.117); with normal controls, the heritability on the observed scale was 1.2% (SE = 0.013, *P* = 0.193), translating to 4.3% on the liability scale (the proportion of cases was 0.085). The results of all analyses combined indicate a small but non-zero heritability for susceptibility to gastrointestinal infections.

### Genome-wide association study

**Fig. 2:**
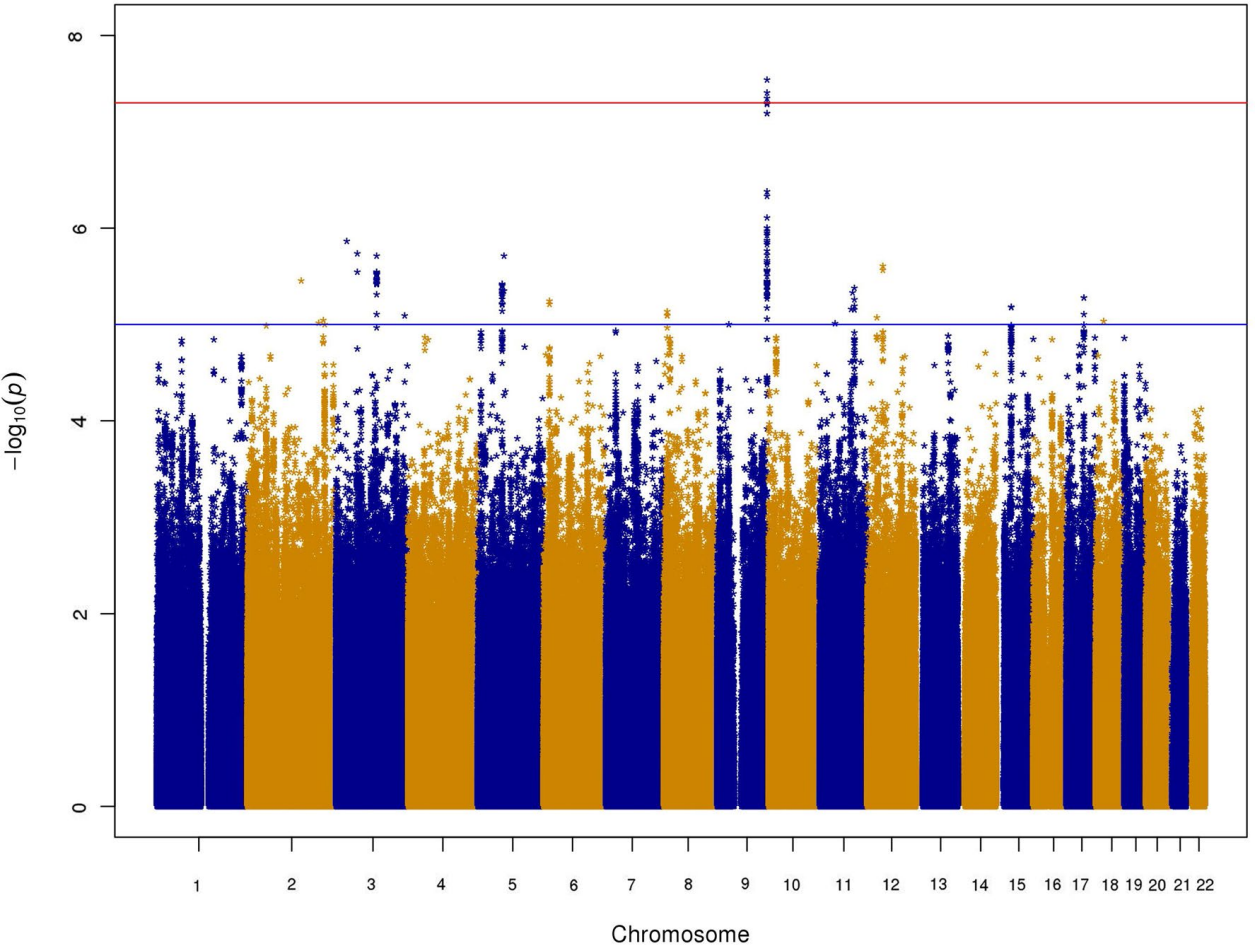
Manhattan plot for the gastrointenstinal infection GWAS. The red line is for the genome-wide significance threshold (5 × 10^–8^), and the blue line is for suggestive association (10^–5^)

**Fig. 3:**
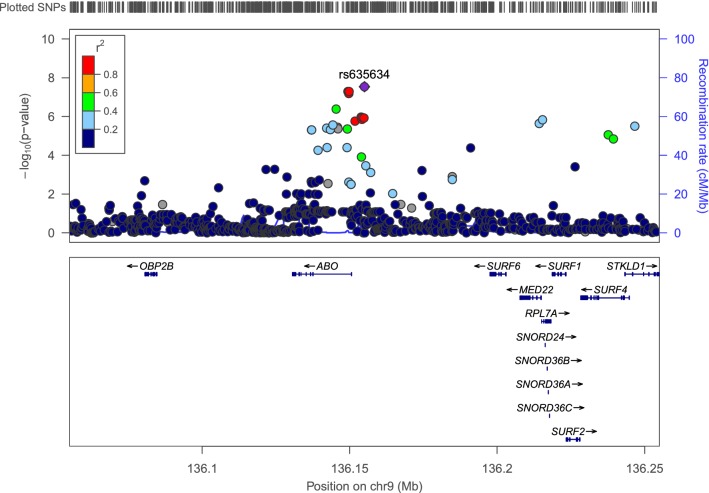
Regional association plot around the top GWAS SNP

Overall, there were 143 significant or suggestive associations (*P* ≤ 10^–5^) (Fig. 2, Supplementary Table S2, Supplementary Figure S1 is the QQ plot, *λ* = 1.02). We discovered one genome-wide significant locus for gastrointestinal infection in which the top SNP was rs635634 (chr9: 136,155,000), with *P* = 2.9 × 10^–8^, odds ratio (OR) = 1.13 relative to the T allele (95% CI: 1.08–1.18) as can be seen in the LocusZoom (Pruim et al. 2010) plot (Fig. 3). This SNP was not genome-wide significant in a GWAS which defined cases as having any infection category and controls as having none (Nudel et al. 2019). A *Z* test for whether the estimate from the gastrointestinal infection GWAS was significantly larger showed that the difference was significant (*Z* = 4.47, *P* = 3.91 × 10^–6^, one sided), suggesting that the association may be more specific to gastrointestinal infections, even though controls had none of the other infections in addition to not having gastrointestinal infections.

## Discussion

In this population-based study, we investigated the genetic architecture of gastrointestinal infections requiring hospitalization among 65,534 unrelated Danish individuals and the associations between gastrointestinal infections and six psychiatric and neurodevelopmental disorders.

Our heritability estimation highlighted a modest but significant contribution of common SNPs to susceptibility to gastrointestinal infections. We also found significant correlations in the comorbidity analyses, suggesting that gastrointestinal infections tend to co-occur with psychiatric and neurodevelopmental disorders, although the cause of this association is not discoverable from this set of analyses. Given the abundance of studies supporting a role for the microbiome in psychiatric and neurodevelopmental disorders, our result may suggest that some genetic factors may underlie both susceptibility to infection and risk of psychiatric or neurodevelopmental disorders, perhaps through the infection mechanism.

One plausible mechanism for the genetic predisposition to gastrointestinal infection is exemplified by our genome-wide significant association between rs635634 and gastrointestinal infections. This SNP has previously been found to be associated with immune markers such as myeloid white cell/neutrophil count (Astle et al. 2016), as well as with tonsillectomy (Pickrell et al. 2016). Furthermore, the same SNP has been associated with conditions such as coronary artery disease (Pickrell et al. 2016), shown suggestive association to ischemic stroke (Traylor et al. 2017), and been connected to metabolic traits such as fasting glucose levels (Wessel et al. 2015). Some of these traits, namely the immune markers, are related to infection and could potentially be on some pathway together with the infection phenotype in our study. We examined the association between rs635634 and myeloid white cell/neutrophil count in the above study and observed that the T allele was associated with reduced levels (in theory, this could make people more prone to having infections). That said, we note again that the SNP did not show genome-wide significant association with general infection in our previous study. Furthermore, we examined the top SNPs associated with the index class “myeloid white cell” in general and neutrophil count (rs10980797 and rs12600856, respectively) in the same study. Neither SNP showed association in our GWAS (OR = 0.9938, 1.0071; *P* = 0.7384, 0.7086, respectively). However, even if the effect of the SNP on the phenotype is through an immune mechanism captured by one of the above markers, it might still not be necessarily appropriate to correct for the latter to assess the total causal effect (Schisterman et al. 2009). Our top SNP was also highlighted in a study of different kinds of infections, although it was only associated with tonsillectomy at a genome-wide level of significance (Tian et al. 2017). Interestingly, it lies close to the *ABO* gene and was reported as an expression quantitative trait locus (eQTL) for the gene in whole blood (*P* = 3.6 × 10^–34^), GTEx Browser, accessed on October 2nd 2019 (GTEx Consortium 2013), which is of particular interest, since connections between blood groups and infections have been observed previously, and the expression of blood groups is connected to the maturation of the gastrointestinal microbiome (Cooling 2015). Pertinent to this study are the associations between the O blood group and susceptibility to gastrointestinal infection with the *H. pylori* bacterium (Boren et al. 1993; Jaff 2011), as well as susceptibility to infection with the Norwalk virus, which causes acute gastroenteritis (Hutson et al. 2002), and the likelihood of having a more severe reaction while infected with the *V. Cholerae* bacterium, which causes cholera (though it should be noted that the likelihood of individuals with the O blood group’s being infected in the first place was lower in that study) (Harris et al. 2005). The O blood group is the manifestation of the lack of the A or B antigens, caused by a deletion in the O allele, which causes a frameshift. In our study, the T allele increased the risk of acquiring a gastrointestinal infection. In GTEx, the same allele is associated with reduced expression of *ABO*, *i.e.* less of antigens A or B in individuals expressing those antigens. This association is also reported by the eQTLGen Consortium (blood eQTLs in over 31,000 individuals, accessed on January 20th 2020) (Võsa et al. 2018), where the T allele has a *Z* score of − 35.6922 (*P* = 5.2235 × 10^–279^). Thus, our results are in line with the suggestion that blood type O, or lower expression of the *ABO* gene and potentially of antigens A or B, in this case, may be associated with gastrointestinal infections. We emphasize that, as we employed a covariate for psychiatric diagnosis in our GWAS, it does not seem to be the case that the link with the *ABO* gene discovered in this study reflects a direct, causal link between blood groups and psychiatric disorders through association with this SNP. In the newly released FinnGen browser (accessed on January 16th 2020), rs635634 showed association with the phenotype “intestinal infectious diseases” (which overlapped almost entirely with ours, based on the included ICD-10 codes), but some differences between our studies should be noted, namely, that the FinnGen GWAS did not include a covariate for psychiatric diagnosis, and indeed many of the individuals included in their study had one e.g., 24,308 on depression medications and 5007 with schizophrenia, schizotypal and delusional disorders, and, furthermore, their analysis used controls which potentially had other types of infections, unlike ours (FinnGen 2020). Nonetheless, the association reported there showed the same trend as in our study, with a beta of − 0.075 for the C allele (*P* = 3.2 × 10^–4^), i.e. the C allele reduced risk, and the T allele consequently increased it.

We have also identified a number of associations which were suggestive but not genome-wide significant (Supplementary Table S2). It is noteworthy to mention that some of those have been found to be associated with other types of infections, even significantly so, in an aforementioned study (Tian et al. 2017). For example, rs687621 (*P* = 4.94 × 10^–6^ in our study) was genome-wide significantly associated with childhood ear infections. Many of the overlaps between the SNPs in Supplementary Table S2 in our study and SNPs highlighted by Tian et al. were with SNPs implicated in childhood ear infections or tonsillectomy in the latter study. However, even though we did have data for otitis media, our top SNP did not show even suggestive association with that infection category or with the other infection categories as described in Supplementary Table S1. These results could imply some pleiotropty, but, since the above study did not have a gastrointestinal infection phenotype similar to ours, and in the absence of further evidence, we cannot make any decisive statement on this possibility.

Some limitations of the study need to be emphasized. The iPSYCH cohort is a relatively young sample, which means that at least some individuals are likely to be diagnosed with infections later on. Moreover, due to the unique nature of the cohort (which includes national registry data from birth on every individual), it is hard to find a suitable replication sample, especially one that is publicly available. We did not have access to an independent sample encompassing phenotypic data on the infection and psychiatric diagnoses from birth and raw genetic data, as used in this study. This means that our results need to be replicated in future studies before they could be considered decisive.

In conclusion, we found significant correlations across several psychiatric and neurodevelopmental disorders and gastrointestinal infections. We have also estimated the contribution of common SNPs to susceptibility to gastrointestinal infections and identified a genome-wide significant association; one allele, independently associated with lower expression levels of *ABO*, increased the risk of having gastrointestinal infections. This result is in line with studies which found that individuals with blood type O are more prone to having this type of infection, and, to our knowledge, it is the first genetic evidence attesting to the relevance of the blood group to susceptibility to this type of infection. Our results may thus constitute one further step towards the understanding of the genetic mechanism for this category of infections. Taken together, our study highlights the importance of a multifaceted and integrative approach to infection research, which used hospital registers, genetics, psychiatry, and epidemiological resources. We hope our study will help raise awareness to the potential links across these disciplines among health practitioners and researchers alike.

## Electronic supplementary material

Below is the link to the electronic supplementary material.

Supplementary Figure S1 (PDF 123 kb)

Supplementary Table S1 (DOCX 16 kb)

Supplementary Table S2 (XLSX 19 kb)

## Data Availability

iPSYCH data are stored in a national HPC facility in Denmark. The iPSYCH initiative is committed to providing access to these data to the scientific community, in accordance with Danish law. Researchers may be granted access upon request to the iPSYCH management. Summary statistics are available from the corresponding author.
