# Supplementary figures and images for: A large population-based investigation into the genetics of susceptibility to gastrointestinal infections and the link between gastrointestinal infections and mental illness
Source: Hum Genet. 2020 Mar 9;139(5):593–604. doi: 10.1007/s00439-020-02140-8 (PMC7170821; doi:10.1007/s00439-020-02140-8)

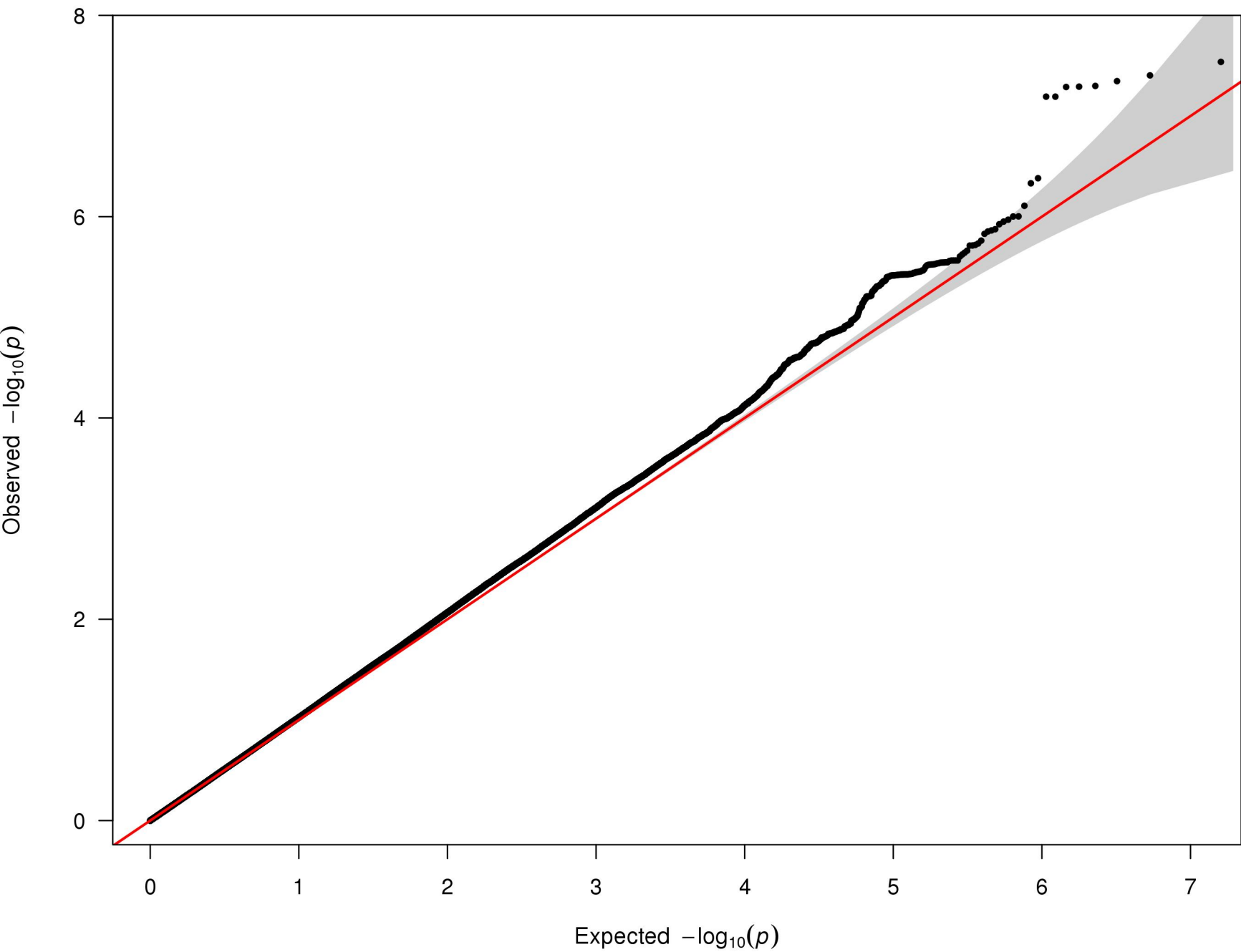

Supplement: Supplementary file 1 — Supplementary Figure S1 (PDF 123 kb) [file 439_2020_2140_MOESM1_ESM.pdf]
